# Contribution to the knowledge of Neanurinae of north-western Iran with description of seven new species (Collembola, Neanuridae)

**DOI:** 10.3897/zookeys.992.56921

**Published:** 2020-11-12

**Authors:** Adrian Smolis, Dariusz Skarżyński

**Affiliations:** 1 Institute of Environmental Biology, Department of Invertebrate Biology, Evolution and Conservation, University of Wrocław, Przybyszewskiego 65, 51-148 Wrocław, Poland University of Wrocław Wrocław Poland

**Keywords:** Asia, new records, springtails, taxonomy, western Palearctic

## Abstract

Seven new species of the subfamily Neanurinae from north-western Iran are described and illustrated in detail. *Endonura agnieskae***sp. nov.** differs from the most similar congener, *E. reticulata* (Axelson, 1905), in chaetotaxic details and the arrangement of tubercles on the dorsal side of the body. *Endonura annae***sp. nov.** can be easily recognised by its wide labrum, the absence of chaetae C on the head and the presence of a toothed claw. *Endonura schwendingeri***sp. nov.** is especially distinctive due to the absence of chaetae A and Ocp on the head and the presence of the male ventral organ. *Deutonura breviseta***sp. nov.** is related and most similar to *D. persica* Smolis, Shayanmehr & Yoosefi-Lafooraki, 2018, described recently and known from Mazandran Province in Iran. The new species can be easily distinguished by the following set of features: dark pigmented body, presence of chaetae C and Dl3 on the head, absence of microchaetae on the furca rudimentary, presence of thickened macrochaetae on dorsal side of body and absence of cryptopygy. The main characteristics of *Deutonura sengleti***sp. nov.** include a white body with dark pigmented eyes, the fusion of tubercles Di and De on the first thoracic segment and the presence of the male ventral organ. *Deutonura iranica***sp. nov.** is superficially similar to *D. gibbosa* Porco, Bedos & Deharveng, 2010, a species known from the Alps and Jura in Europe, but it differs in the body colour and the number of labial chaetae and chaetae (L+So) on the head. *Paravietnura rostrata***sp. nov.**, the first member of this enigmatic and intriguing genus known from Iran, is characterised by an unusually elongate ogival labrum and extreme reduction of dorsal chaetotaxy. Furthermore, new records of several other species of the subfamily: *Cryptonura maxima* Smolis, Falahati & Skarżyński, 2012; *C. persica* Smolis, Falahati & Skarżyński, 2012; *Deutonura persica*; *Endonura longirostris* Smolis, Shayanmehr, Kuznetsova & Yoosefi-Lafooraki, 2017; *E. paracentaurea* Smolis, Shayanmehr, Kuznetsova & Yoosefi-Lafooraki, 2017; *Neanura deharvengi* Smolis, Shayanmehr & Yoosefi-Lafooraki, 2018; *N. muscorum* (Templeton, 1835) and *Protanura papillata* Cassagnau & Delamare Deboutteville, 1955 are given. The present study is based on the rich material collected by Antoine Senglet and loaned by Peter J. Schwendinger.

## Introduction

Springtails, classified within the subfamily Neanurinae, differ significantly in terms of morphology and behaviour from other Collembola. First of all, they have completely lost the furcula and their movement may be defined as exceptionally slow compared to the majority of springtails. Another noticeable difference between them and the majority of other Collembola is the covering of the dorsal and lateral sides of the body by spherical structures naming tubercles, which make them resemble a mulberry. In addition, chaetae covering Neanurinae body are usually strongly developed, elongated and considerably widened, as well as covered with numerous teeth (Deharveng 1983; Smolis 2008). Paradoxically, although they do not have a furcula, i.e. structures enabling express escape from predators, Neanurinae are an example of an evolution success, demonstrated by its over 800 currently described taxa which constitutes nearly one tenth of all the known Collembola (Bellinger et al. 2020; Smolis and Greenslade 2020). Regarding the actual distribution of the subfamily, the largest species diversity is observed both in tropical and temperate forests on all continents, excluding Antarctica (i.e. Yosii 1976; Cassagnau and Deharveng 1984; Deharveng and Weiner 1984; Cassagnau 1988; Deharveng 1989; Deharveng and Bedos 1992; Cassagnau 1996; Deharveng and Suhardjono 2000; Palacios-Vargas and Simón Benito 2007; Zhi-Chun and Jian-Xiu 2008; Palacios-Vargas and Deharveng 2014; Smolis and Deharveng 2015; Luo and Palacios-Vargas 2016; Ji-Gang et al. 2018). Nevertheless, knowledge on global diversity of the subfamily is still insufficient and far from complete as many areas, i.e. the Middle East, North Africa, New Guinea or Central Asia, are poorly surveyed in this respect.

An examination of an exceptionally-rich material of Neanurinae from north-western Iran (Provinces: Gilan, Golestan, Kermanshah, Mazandaran, North Khorasan, Semnan and West Azerbaijan), collected in the early 1970s by Antoine Senglet and loaned for the presented studies by Peter J. Schwendinger (curator of the Muséum d’histoire naturelle in Geneva, Switzerland), has revealed seven unknown species of this subfamily. Their detailed and illustrated descriptions are provided with new records of several other known species classified to Neanurinae.

## Materials and methods

The specimens were cleared in Nesbitt’s fluid, subsequently mounted on slides in Swan’s medium and studied using a Nikon Eclipse E600 phase contrast microscope. Figures were drawn with a camera lucida and prepared for publication using Adobe Photoshop CS3.

The whole material, types as well as the other material, is deposited in the Muséum d’histoire naturelle in Geneva, Switzerland.

### Terminology

Terminology and layout of the tables used in the paper follow Deharveng (1983), Deharveng and Weiner (1984), Smolis and Deharveng (2006) and Smolis (2008).

### Abbreviations

General morphology:

**Abd.** abdomen;

**Ant.** antenna;

**AOIII** sensory organ of antennal segment III;

**Cx** coxa;

**Fe** femur;

**Scx2** subcoxa 2;

**T** tibiotarsus;

**Th.** thorax;

**Tr** trochanter;

**VT** ventral tube.

Groups of chaetae:

**Ag** antegenital;

**An** chaetae of anal lobes;

**Ap** apical;

**Ca** centroapical;

**Cm** centromedial;

**Cp** centroposterior;

**D** dorsal;

**Fu** furcal;

**Vc** ventrocentral;

**Ve**or**ve** ventroexternal;

**Vea** ventroexternoanterior;

**Vem** ventroexternomedial;

**Vep** ventroexternoposterior;

**Vel** ventroexternolateral;

**Vec** ventroexternocentral;

**Vei** ventroexternointernal;

**Vi**or**vi** ventrointernal;

**Vl** ventrolateral.

Tubercles:

**Af** antenno-frontal;

**Cl** clypeal;

**De** dorsoexternal;

**Di** dorsointernal;

**Dl** dorsolateral;

**L** lateral;

**Oc** ocular;

**So** subocular.

Types of chaetae:

**Ml** long macrochaeta;

**Mc** short macrochaeta;

**Mcc** very short macrochaeta;

**Me** mesochaeta;

**mi** microchaeta;

**ms** s-microchaeta;

**S**or**s** chaeta s;

**Bs** s-chaeta on Ant. IV;

**miA** microchaetae on Ant. IV;

**iv** ordinary chaetae on ventral Ant. IV;

**or** organite of Ant. IV;

**brs** border s-chaeta on Ant. IV;

**i** ordinary chaeta on Ant. IV;

**mou** cylindrical s-chaetae on Ant. IV (“soies mousses”);

**x** labial papilla x;

**L**’ ordinary lateral chaeta on Abd. V;

**B4**, **B5** ordinary chaetae on tibiotarsi.

## Taxonomy

### Endonura agnieskae sp. nov.

Taxon classificationAnimaliaPoduromorphaNeanuridae

6A4AAFB9-E507-5ADE-9D56-17DB127BFCDC

http://zoobank.org/B8FE7E36-B1F9-4D2E-BA23-1588CCA1126D

Figs 1–13 , Tables 1 , 2 , 3

#### Type material.

***Holotype***: adult female on slide, Iran, Mazandaran Province, Nashtarud, forest reserve, sifting, 10.VII.1973, leg. A. Senglet, sample 7318. ***Paratypes***: 4 females, 2 males and 2 juveniles on slide, same data as holotype.

#### Other material.

Female on slide, Iran, Mazandaran Province, Kiasar (36°16'N, 53°25'E), 10.VII.1975, leg. A. Senglet, 7546; 9 females, 2 males and juvenile on slide, Gilan Province, Limir, large trees in marsh, sifting, 28.VI.1973, leg. A. Senglet, 7306; female on slide, Iran, Gilan Province, Paresar, tree holes, leaves, sifting, 2.VII.1973, leg. A. Senglet, 7310; female on slide, Gilan Province, road to Jirandeh, 1000 m a.s.l., forest, 9.VIII.1974, leg. A. Senglet, 7486; female on slide, Semnan Province, near Loveh (37°19'N, 55°46'E / 1300 m a.s.l.), 22.VIII.1975, leg. A. Senglet, 7574.

**Table 1.** Chaetotaxy of *Endonura agnieskae* sp. nov.: Cephalic chaetotaxy–dorsal side.

Tubercle	Number of chaetae	Types of chaetae	Names of chaetae
Cl	4	Ml	F
		me	G
Af	11	Ml	B
		Mc	A, O, C, D, E
Oc	3	Ml	Ocm
		Mc	Ocp
		mi	Oca
Di	2	Ml	Di1
		Mcc	Di2
De	2	Ml	De1
		Mcc	De2
Dl	6	Ml	Dl5, Dl1
		Mc	Dl3
		Mcc	Dl2, Dl4, Dl6
(L+So)	10	Ml	L1, L4, So1
		Mcc	L2
		mi	L3, So2
		me	So3–6

#### Etymology.

The new species is dedicated to Agnieszka, wife of the first author.

#### Diagnosis.

Habitus typical of the genus *Endonura*. Dorsal tubercles present and well developed. 2+2 large pigmented eyes. Buccal relatively short, labrum nonogival. Central area of head with complete chaetotaxy. Tubercles Cl and Af separate. Tubercles Dl and (L+So) on head with 6 and 10 chaetae, respectively. Tubercles Di on Th. I present and fused with tubercle De. Tubercles De on Th. II and III with 3 and 4 chaetae, respectively. Tubercles L on Abd. III and IV with 3–4 and 7 chaetae, respectively. Abd. IV and V with 8 and 3 tubercles, respectively. Furcal rest without mi. Claw without inner tooth. Tibiotarsi with chaetae B4 and B5 rather short.

**Figures 1–13.**
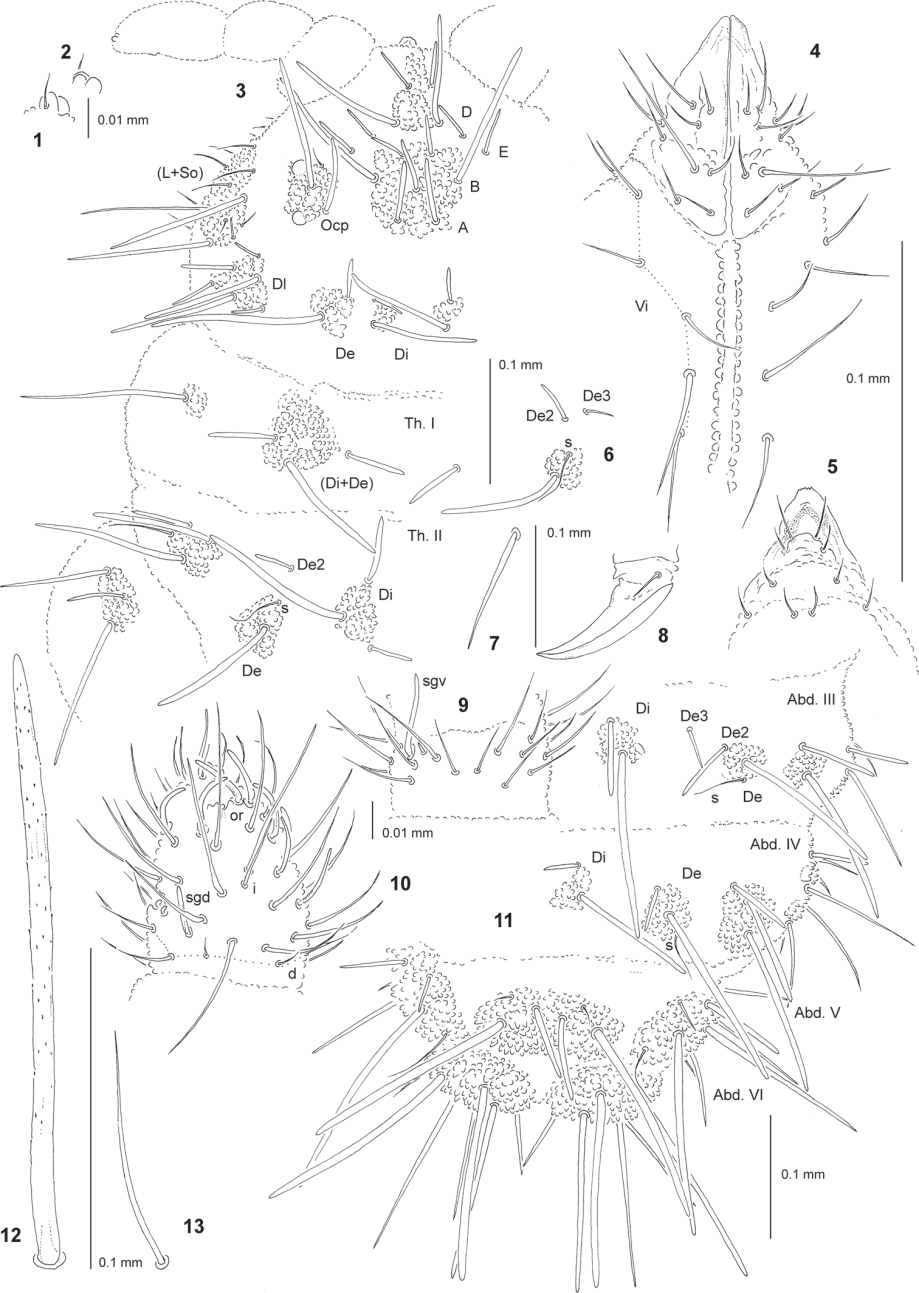
*Endonura agnieskae* sp. nov.: **1** apical bulb, dorsal view **2** apical bulb, ventral view **3** chaetotaxy of head and Th., dorsolateral view **4** chaetotaxy of labium and group Vi (holotype) **5** chaetotaxy and ventral sclerifications of labrum (holotype) **6** tubercle De of Th. III **7** chaeta B4 of leg III **8** claw of leg III, lateral view **9** ventral chaetotaxy of Ant. III **10** dorsal chaetotaxy of Ant. III–IV (holotype) **11** dorsal chaetotaxy of Abd. III–VI **12** chaeta Di1 of Abd. V **13** sensillum of Abd. V.

#### Description.

General. Body length (without antennae): 0.8 (juvenile) to 1.7 mm (holotype: 1.5 mm). Colour of the body bluish-grey. 2+2 large black eyes, in a typical arrangement for the genus (one anterior and one posterior eye, Fig. 3).

Chaetal morphology. Dorsal ordinary chaetae of five types: long macrochaetae (Ml), short macrochaetae (Mc), very short macrochaetae (Mcc), mesochaetae and microchaetae. Long macrochaetae thick, slightly arc-like or straight, narrowly sheathed, feebly serrated, apically rounded (Figs 3, 6, 11, 12). Macrochaetae Mc and Mcc morphologically similar to long macrochaetae, but much shorter. Mesochaetae similar to ventral chaetae, thin, smooth and pointed. Microchaetae similar to mesochaetae, but clearly shorter. S-chaetae of terga thin, smooth and short, distinctly shorter than nearby macrochaetae (Figs 3, 6, 11, 13).

Antennae. Typical of the genus. Dorsal chaetotaxy of Ant. III–IV as Fig. 10 and Table 2. S-chaetae of Ant. IV of medium length and moderately thickened (Fig. 10). Apical vesicle distinct, trilobate (Figs 1 and 2). Ventral chaetotaxy of Ant. III–IV as Fig. 9 and Table 2, sensillum sgv long and slightly s-shaped.

**Table 2.** Chaetotaxy of *Endonura agnieskae* sp. nov.: Chaetotaxy of antennae.

Segment, Group	Number of chaetae	Segment, Group	Number of chaetae adult
I	7	IV	or, 8 S, i, 12 mou, 6 brs, 2 iv
II	12		
III	5 sensilla AO III		
ve	5	ap	8 bs, 5 miA
vc	4	ca	2 bs, 3 miA
vi	4	cm	3 bs, 1 miA
d	5	cp	8 miA, 1 brs

Mouthparts. Buccal cone rather short with labral sclerifications nonogival. Labrum chaetotaxy: 4/2, 4 (Fig. 5). Labium with four basal, three distal and three lateral chaetae, papillae x absent (Fig. 4). Maxilla styliform, mandible thin and tridentate.

Dorsal chaetotaxy and tubercles. Chaetotaxy of head complete (Fig. 3). Tubercles Di on head present, on Th. I differentiated and fused with De. Th. III and Abd. I–III with chaetae De3 free (Figs 6 and 11). On Abd. I–III, the line of chaetae De1–chaeta s parallel to the dorsomedian line (Fig. 11). On Abd. IV chaetae Di1 short. Cryptopygy absent, Abd. VI well visible from above. Chaeta Di2 on Abd. V as Mc, Mccormi.

Ventral chaetotaxy. On head, groups Vea, Vem and Vep with 3, 3–4, 4 chaetae, respectively. Group Vi on head with 6 chaetae (Fig. 4). On Abd. IV, furca rudimentary without microchaetae. On Abd. IV, tubercle L without free chaeta.

Legs. Chaetotaxy of legs as in Table 3. Claw without internal tooth (Fig. 8). On tibiotarsi, chaeta M present and chaetae B4 and B5 rather short and pointed (Fig. 7).

**Table 3.** Chaetotaxy of *Endonura agnieskae* sp. nov.: Postcephalic chaetotaxy.

	Terga				Legs				
	Di	De	Dl	L	Scx2	Cx	Tr	Fe	T
Th. I	3		1	–	0	3	6	13	19
Th. II	3	2+s	3+s+ms	3	2	7	6	12	19
Th. III	3	3+s	3+s	3	2	8	6	11	18
					Sterna				
Abd. I	2	3+s	2	3	VT: 4				
Abd. II	2	3+s	2	3	Ve: 5; chaeta Ve1 present				
Abd. III	2	3+s	2	3	Vel:5–6; Fu: 5 me, 0 mi				
Abd. IV	2	2+s	3	5–6	Vel: 4; Vec: 2; Vei: 2; Vl: 4				
Abd. V	(3+3)	7–8+s			Ag: 3; Vl: 1				
Abd. VI		7			Ve: 14; An: 2 mi				

#### Remarks.

Due to the general appearance, dorsal and ventral chaetotaxy, *E. agnieskae* sp. nov. strongly resembles *E. reticulata* (Axelson, 1905), Holarctic and circumboreal species occurring in tundra, boreal and temperate biotopes of northern Europe (Scandinavian Peninsula), north-eastern Asia and North America (Smolis et al. 2011). Nevertheless, these species can be easily distinguished from each other by the set of characters: size of the eyes (expressed by the ratio of anterior eye diameter and diameter of base of chaeta Ocm, in *agnieskae* 2:1, in *reticulata* 1:1 or 5:4), the number of lateral labial chaetae (in *agnieskae* three, in *reticulata* four), the length of chaetae Ocp and A on the head (in *agnieskae*, equal in length, in reticulata chaeta Ocp, longer than chaeta A), the presence of tubercle Di on Th. I (in *agnieskae*, present and fused with De, in *reticulata*, absent), the location of chaeta De2 on Abd. I–III (in *agnieskae*, connected with tubercle De, in *reticulata*, free), the location of chaeta s on Abd. I–III (in *agnieskae*, the line of chaetae De1–chaeta s parallel to the dorsomedian line, in *reticulata*, not parallel) and the length of chaeta Di1 on Abd. IV (in *agnieskae*, distinctly shorter than chaeta Di1 on Abd. III, in *reticulata*, longer or equal to chaeta Di1 on Abd. III).

### Endonura annae sp. nov.

Taxon classificationAnimaliaPoduromorphaNeanuridae

1D2FEFC0-529D-5E0B-853C-606294388B27

http://zoobank.org/A26E5348-95D7-4E84-BB9A-C50383757D11

Figs 14–27 , Tables 4 , 5 , 6

#### Type material.

***Holotype***: adult female on slide, Iran, Gilan Province, road to Dyavaherdeh, 1100–1300 m a.s.l., 7.VIII. 1974, leg. A. Senglet, sample 7484. ***Paratypes***: 2 females, male and juvenile on slide, same data as holotype.

#### Other material.

Iran, 7 females and male on slide, Gilan Province, near Asalem, 300–600 m a.s.l., large beeches, sifting, 30.VI.1973, leg. A. Senglet, 7308; 3 females on slide, Gilan Province, Shahrbijar, tree hole, humus, sifting, 6.IX.1973, leg. A. Senglet, 7366; 4 females and juvenile on slide, Gilan Province, Asalem (37°45'N, 48°57'E), leaves and tree holes, sifting, 11.VI.1975, leg. A. Senglet, 7519; juvenile on slide, Mazandaran Province, Pol-e Zanguleh, 2300 m a.s.l., 12.VII.1973, leg. A. Senglet, 7320.

#### Etymology.

The new species is dedicated to Anna, wife of the second author.

#### Diagnosis.

Habitus typical of the genus *Endonura*. Dorsal tubercles present and well developed. 2+2 large pigmented eyes. Buccal cone short, labrum nonogival. Head with chaetae A, B, D and E. Chaetae O and C absent. Tubercles Cl and Af separate. Tubercles Dl and (L+So) on head with 6 and 8 chaetae, respectively. Tubercles Di on Th. I present. Tubercles De on Th. II and III with 3 and 4 chaetae, respectively. Tubercles L on Abd. III and IV with 3 and 6 chaetae, respectively. Abd. IV and V with 8 and 3 tubercles, respectively. Furcal rest without mi. Claw with inner tooth. Tibiotarsi with chaetae B4 and B5 rather short.

#### Description.

**Figures 14–27.**
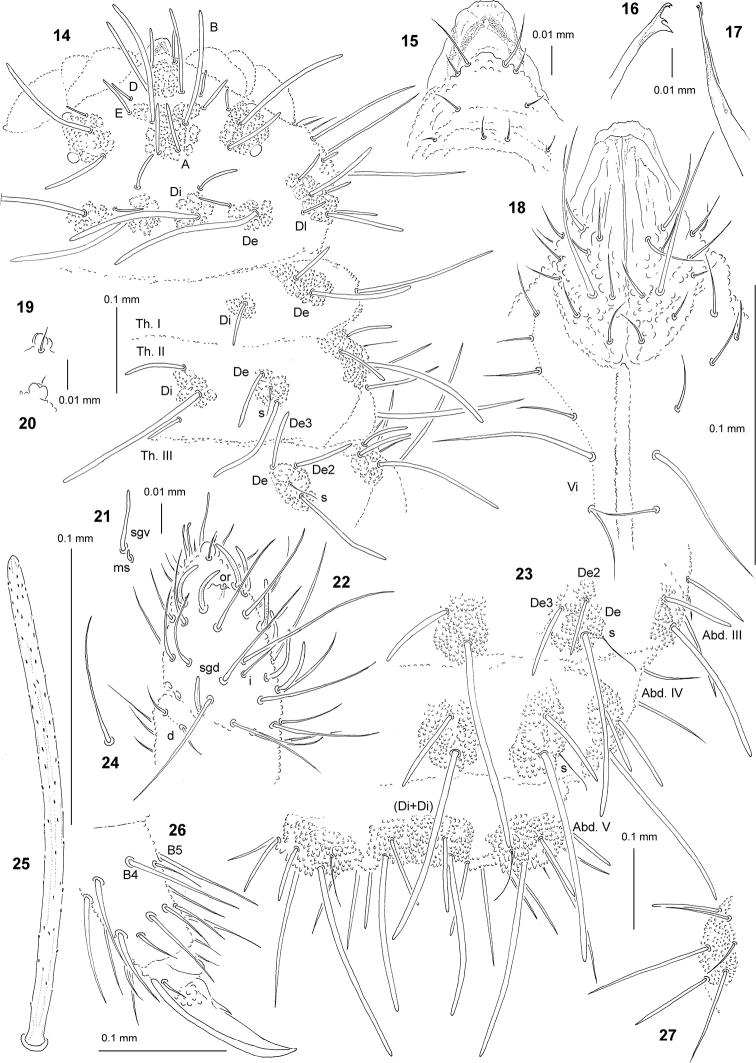
*Endonura annae* sp. nov.: **14** chaetotaxy of head and Th. (holotype), dorsolateral view **15** chaetotaxy and ventral sclerifications of labrum **16** Mandible **17** Maxilla **18** chaetotaxy of labium and group Vi**19** apical bulb, dorsal view **20** apical bulb, ventral view **21** sensillum sgv and microsensillum of Ant. III **22** dorsal chaetotaxy of Ant. III–IV **23** dorsal chaetotaxy of Abd. III–VI (holotype) **24** sensillum of Abd. V **25** chaeta Di1 of Abd. V **26** tibiotarsus and claw of leg III, lateral view **27** tubercle L of Abd. IV.

General. Body length (without antennae): 0.8 to 1.45 mm (holotype: 1.25 mm). Colour of the body white. 2+2 large black eyes, in a typical arrangement for the genus (Fig. 14).

Chaetal morphology. Dorsal ordinary chaetae of four types: long macrochaetae (Ml), short macrochaetae (Mc), very short macrochaetae (Mcc) and mesochaetae. Long macrochaetae thick, slightly arc-like, narrowly sheathed, feebly serrated, apically rounded (Figs 14, 23, 25). Macrochaetae Mc and Mcc morphologically similar to long macrochaetae, but shorter. Mesochaetae similar to ventral chaetae, thin, smooth and pointed. S–chaetae of terga thin, smooth and short, notably shorter than nearby macrochaetae (Figs 14, 23, 24).

**Table 4.** Chaetotaxy of *Endonura annae* sp. nov.: Cephalic chaetotaxy–dorsal side.

Tubercle	Number of chaetae	Types of chaetae	Names of chaetae
Cl	4	Ml	F
		me	G
Af	8	Ml	B
		Mc	A, E
		Mcc	D
Oc	3	Ml	Ocm
		Mc	Ocp
		Mcc	Oca
Di	2	Ml	Di1
		Mc	Di2
De	2	Ml	De1
		Mcc	De2
Dl	6	Ml	Dl5, Dl1
		Mc	Dl4
		Mcc	Dl2, Dl3, Dl6
(L+So)	8	Ml	L1, L4, So1
		Mcc	L2
		me	So3–6

Antennae. Typical of the genus. Dorsal chaetotaxy of Ant. III–IV as Fig. 22 and Table 5. S-chaetae of Ant. IV of medium length and moderately thickened, sensillum sgd notably short (Fig. 22). Ant. III with two chaetae d. Apical vesicle distinct, trilobate (Figs 19, 20). Ventral chaetotaxy of Ant. III as in Table 5, sensillum sgv long and slightly s-shaped (Fig. 21).

**Table 5.** Chaetotaxy of *Endonura annae* sp. nov.: Chaetotaxy of antennae.

Segment, Group	Number of chaetae	Segment, Group	Number of chaetae adult
I	7	IV	or, 8 S, i, 12 mou, 6 brs, 2 iv
II	12		
III	5 sensilla AO III		
ve	5	ap	8 bs, 5 miA
vc	4	ca	2 bs, 3 miA
vi	4	cm	3 bs, 1 miA
d	2	cp	8 miA, 1 brs

Mouthparts. Buccal short and wide with labral sclerifications nonogival (Fig. 15). Labrum chaetotaxy: 4/2, 4 (Fig. 15). Labium with four basal, three distal and four lateral chaetae, papillae x absent (Fig. 18). Maxilla styliform (Fig. 17), mandible with four teeth and relatively thin (Fig. 16).

Dorsal chaetotaxy and tubercles. Head without chaetae O, C, So2 and L3 (Fig. 14). Chaetae D free and not connected with tubercle. Tubercles Di on Th. I differentiated, not fused with tubercles De (Fig. 14). Th. III and Abd. I–III without free chaetae De2 and De3 (Figs 14, 23). On Abd. I–III, the line of chaetae De1–chaeta s perpendicular to the dorsomedian line. On Abd. III–IV, chaetae Di1 notably longer than chaetae Di1 of Abd. V (Fig. 23). On Abd. V, tubercle (Di+Di) with 2+2 chaetae. Cryptopygy strongly developed, Abd. VI practically not visible from above (Fig. 23).

Ventral chaetotaxy. On head, groups Vea, Vem and Vep with 3, 4, 4 chaetae, respectively. Group Vi on head with 6 chaetae (Fig. 18). On Abd. IV, furca rudimentary without macrochaetae, tubercle L with 6 chaetae (Fig. 27). On Abd. V, chaetae Vl and L’ present.

Legs. Chaetotaxy of legs as in Table 6. Claw with internal tooth. On tibiotarsi, chaeta M present and chaetae B4 and B5 relatively short and pointed (Fig. 26).

**Table 6.** Chaetotaxy of *Endonura annae* sp. nov.: Postcephalic chaetotaxy.

	Terga				Legs				
	Di	De	Dl	L	Scx2	Cx	Tr	Fe	T
Th. I	1	2	1	–	0	3	6	13	19
Th. II	3	2+s	3+s+ms	3	2	7	6	12	19
Th. III	3	3+s	3+s	3	2	8	6	11	18
					Sterna				
Abd. I	2	3+s	2	3	VT: 4				
Abd. II	2	3+s	2	3	Ve: 5; chaeta Ve1 present				
Abd. III	2	3+s	2	3	Vel: 5; Fu: 4–5 me, 0 mi				
Abd. IV	2	2+s	3	6	Vel: 4; Vec: 2; Vei: 2; Vl: 4				
Abd. V	(2+2)	5+s			Ag: 3; Vl: 1, L‘: 1				
Abd. VI		7			Ve: 13–14; An: 2 mi				

#### Remarks.

Morphologically, *E. annae* sp. nov. is strongly reminiscent of *E. persica* Smolis, Kahrarian, Piwnik & Skarżyński, 2016, taxon described from Kermanshah Province in northern Iran (Smolis et al. 2016a). Nevertheless, the new species can be easily recognised by several characters, including: the absence of chaeta C on the head (in *persica* present), the presence of 6 chaetae Dl on the head (in *persica* 5), wide and short buccal cone (in *persica* narrow and long), chaetae E on the head connected with tubercle Af (in *persica* free), chaetae De2 and De3 on Th. II–III, connected with tubercle De (in *persica* free), 2+2 chaetae Di on Abd. V (in *persica* 3+3) and strong cryptopygy (in *persica*, slightly developed).

*E. annae* sp. nov. is also similar to two species with toothed claw: *E. dentifera* Smolis, Skarżyński, Pomorski & Kaprus’, 2007 and *E. dobrolyubovae* Smolis & Kuznetsova, 2018, described from the Crimea and the Caucasus, respectively (Smolis et al. 2007; Smolis and Kuznetsova 2018). These species differ, however, in a number of details: the shape of the buccal cone (in *annae*, wide and short, in *dentifera* and *dobrolyubovae*, narrow and relatively long), the presence of chaeta C on the head (in *annae*, absent, in *dentifera* and *dobrolyubovae*, present), the presence and location of chaeta E on the head (in *annae*, present and connected with tubercle Af, in *dentifera*, present and free, in *dobrolyubovae*, absent), the number of chaetae (L+So) on the head (in *dentifera*, 10 chaetae, in *annae* and *dobrolyubovae*, 8 chaetae), the presence of tubercle Di on Th. I (in *annae*, present, in *dentifera* and *dobrolyubovae*, absent), the location of chaetae De3 on Th. III and Abd. I–III (in *annae*, connected with tubercle De, in *dentifera* and *dobrolyubovae*, free), the presence of male ventral organ (in *annae* and *dentifera*, absent, in *dobrolyubovae*, present) and the presence of cryptopygy (in *annae*, present, in *dentifera* and *dobrolyubovae*, absent).

### Endonura schwendingeri sp. nov.

Taxon classificationAnimaliaPoduromorphaNeanuridae

CF02EA0E-092F-5015-A6E6-BF2206B3C027

http://zoobank.org/19378FF6-D560-4C9E-A729-B7681C695986

Figs 28–41 , Tables 7 , 8 , 9

#### Type material.

***Holotype***: female on slide, Iran, Gilan Province, Paresar, tree holes, leaves, sifting, 2.VII.1973, leg. A. Senglet, sample 7310. ***Paratypes***: 3 females and male on slide, same data as holotype.

#### Other material.

Iran, 3 females and male on slide, Gilan Province, Lunak, 600 m a.s.l., forest, leaves, trunk, sifting, 6.VII.1973, leg. A. Senglet, 7313.

#### Etymology.

The new species is dedicated to Peter J. Schwendinger, curator of the Muséum d’histoire naturelle in Geneva and prominent Austrian Arachnologist.

#### Diagnosis.

Habitus typical of the genus *Endonura*. Dorsal tubercles present. 2+2 large pigmented eyes. Buccal cone relatively long, labrum nonogival. Head with chaetae B, C and D. Chaeta O absent. Tubercles Cl and Af separate. Tubercles Dl and (L+So) on head with 5 and 7 chaetae, respectively. Tubercles Di on Th. I absent. Tubercles De on Th. II and III with 3 and 4 chaetae, respectively. Tubercles L on Abd. III and IV with 2 and 4 chaetae, respectively. Abd. IV and V with 8 and 3 tubercles, respectively. Furcal rest without mi. Claw with inner tooth. Tibiotarsi with chaetae B4 and B5 long.

#### Description.

General. Body length (without antennae): 0.5 (juvenile) to 1.15 mm (holotype: 1.1 mm). Colour of the body bluish-grey. 2+2 large black eyes, in a typical arrangement for the genus (Fig. 29).

**Figures 28–41.**
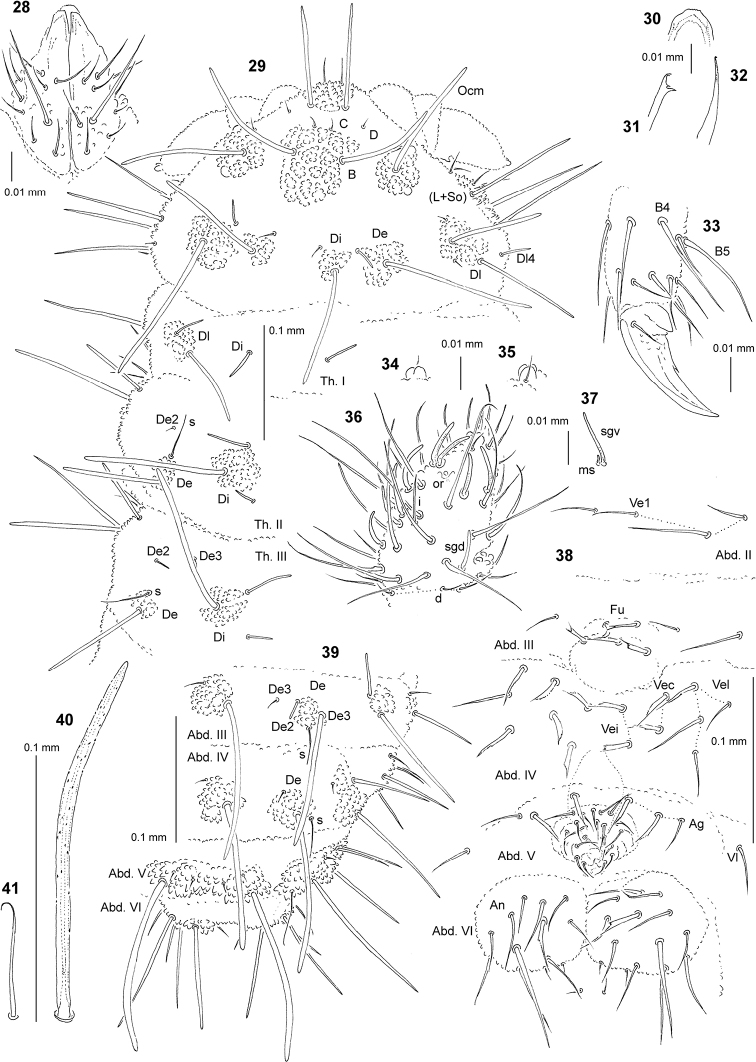
*Endonura schwendingeri* sp. nov.: **28** chaetotaxy of labium **29** chaetotaxy of head and Th. (holotype), dorsolateral view **30** apical part of labrum **31** Mandible **32** Maxilla **33** tibiotarsus and claw of leg III, lateral view **34** apical bulb, ventral view **35** apical bulb, dorsal view **36** dorsal chaetotaxy of Ant. III–IV **37** sensillum sgv and microsensillum of Ant. III **38** ventral chaetotaxy of Abd. II–VI (adult male) **39** dorsal chaetotaxy of Abd. III–VI **40** chaeta Di1 of Abd. V **41** sensillum of Abd. V.

Chaetal morphology. Dorsal ordinary chaetae of five types: long macrochaetae (Ml), short macrochaetae (Mc), very short macrochaetae (Mcc), mesochaetae and microchaetae. Long macrochaetae relatively thin, straight or slightly arc-like, narrowly sheathed, feebly serrated, apically rounded (Figs 29, 39, 40). Macrochaetae Mc and Mcc morphologically similar to long macrochaetae, but much shorter (Figs 29, 39). Mesochaetae similar to ventral chaetae, thin, smooth and pointed. Microchaetae similar to mesochaetae, but clearly shorter (Figs 29, 39). S–chaetae of terga thin, smooth and short, notably shorter than nearby macrochaetae (Figs 29, 39, 41).

**Table 7.** Chaetotaxy of *Endonura schwendingeri* sp. nov.: Cephalic chaetotaxy–dorsal side.

**Tubercle**	**Number of chaetae**	**Types of chaetae**	**Names of chaetae**
Cl	4	Ml	F
		me	G
Af	6	Ml	B
		mi	C, D
Oc	2	Ml	Ocm
		mi	Oca
Di	2	Mc	Di1
		mi	Di2
De	2	Ml	De1
		Mcc	De2
Dl	5	Ml	Dl5, Dl1
		Mcc	Dl4
		mi	Dl2, Dl6
(L+So)	7	Ml	L1, L4, So1
		me	So3–6

**Table 8.** Chaetotaxy of *Endonura schwendingeri* sp. nov.: Chaetotaxy of antennae.

Segment, Group	Number of chaetae	Segment, Group	Number of chaetae adult
I	7	IV	or, 8 S, i, 12 mou, 6 brs, 2 iv
II	12		
III	5 sensilla AO III		
ve	5	ap	8 bs, 5 miA
vc	4	ca	2 bs, 3 miA
vi	4	cm	3 bs, 1 miA
d	5	cp	8 miA, 1 brs

Antennae. Typical of the genus. Dorsal chaetotaxy of Ant. III–IV as Fig. 36 and Table 8. S–chaetae of Ant. IV of medium length and thickened, sensillum sgd short and straight (Fig. 36). Apical vesicle distinct, trilobate (Figs 34, 35). Ventral chaetotaxy of Ant. III–IV Table 8, sensillum sgv as Fig. 37.

Mouthparts. Buccal cone relatively short with labral sclerifications nonogival (Fig. 30). Labrum chaetotaxy: 4/2, 4. Labium with four basal, three distal and three lateral chaetae, papillae x absent (Fig. 28). Maxilla styliform (Fig. 32), mandible relatively thin with two basal and two apical teeth (Fig. 31).

Dorsal chaetotaxy and tubercles. Head without chaetae A, E, Ocp, Dl3, So2, L2 and L3 absent (Fig. 29), chaeta D free. Tubercles Di on Th. I not differentiated (Fig. 29). On Th. III chaetae De2 and De3 free, on Abd. I–III chaetae De3 free (Figs 29, 39). On Abd. I–III, the line of chaetae De1–chaeta s non perpendicular to the dorsomedian line. Cryptopygy present, but weakly developed, Abd. VI partially visible from above (Fig. 39).

Ventral chaetotaxy. On head, groups Vea, Vem and Vep with 3, 4 and 4 chaetae, respectively. Group Vi on head with 6 chaetae. On Abd. IV, furca rudimentary without microchaetae (Fig. 38). On Abd. IV, group L without free chaeta. On Abd. V, chaetae Vl present, chaetae L’ absent (Fig. 38). Male with thick and forked chaetae (male ventral organ) on anal plates (Abd. VI) and in groups: Ag (Abd. V); Vei, Vec and Vel (Abd. IV) and Fu (Abd. III) (Fig. 38).

Legs. Chaetotaxy of legs as in Table 9. Claw with internal tooth. On tibiotarsi, chaeta M present and chaetae B4 and B5 relatively long and pointed (Fig. 33).

**Table 9.** Chaetotaxy of *Endonura schwendingeri* sp. nov.: Postcephalic chaetotaxy.

	Terga				Legs				
	Di	De	Dl	L	Scx2	Cx	Tr	Fe	T
Th. I	1	2	1	–	0	3	6	13	19
Th. II	3	2+s	3+s+ms	3	2	7	6	12	19
Th. III	3	3+s	3+s	3	2	8	6	11	18
					Sterna				
Abd. I	2	3+s	2	2	VT: 4				
Abd. II	2	3+s	2	2	Ve: 4–5; chaeta Ve1 present				
Abd. III	2	3+s	2	2	Vel: 3–4; Fu: 5 me, 0 mi				
Abd. IV	2	2+s	3	4	Vel: 4; Vec: 2; Vei: 2; Vl: 4				
Abd. V	(3+3)	5+s			Ag: 3; Vl: 1				
Abd. VI		7			Ve: 11–12; An: 2 mi				

#### Remarks.

Since *E. schwendingeri* sp. nov. is characterised by chaetotaxic features unknown in other members of the genus, for example, the absence of chaetae A and Ocp on the head, its closer affinities with other *Endonura* species are currently uncertain and hard to assess. However, taking into account the weak development of tuberculation, delicate buccal cone and the presence of well-developed male ventral organ, the new species seems to be most similar to *E. quadriseta* Cassagnau & Péja, 1979, a form shortly described from Greece (Cassagnau and Péja 1979), but recently re-described, based on types and a new material from the Crimea (Smolis et al. 2007). Nevertheless, besides characters mentioned above, these taxa differ in numerous features: the number of lateral labial chaetae (in *schwendingeri*, three, in *quadriseta*, four), the presence of chaetae C and O on the head (in *schwendingeri*, absent, in *quadriseta*, present), the number of chaetae (L+So) on the head (in *schwendingeri*, 7, in *quadriseta*, 9), the number of chaetae Dl on the head (in *schwendingeri*, 5, in *quadriseta*, 6), the number of chaetae L on Abd. III and IV (in *schwendingeri*, 2 and 4, in *quadriseta*, 4 and 7) and the presence of an internal tooth on claws (in *schwendingeri*, present, in *quadriseta*, absent).

### Deutonura breviseta sp. nov.

Taxon classificationAnimaliaPoduromorphaNeanuridae

D04DCB32-1579-58B7-BF3B-BC723607FC14

http://zoobank.org/98297969-EFC7-42C8-9597-14ED4612CD03

Figs 42–52 , Tables 10 , 11 , 12

#### Type material.

***Holotype***: male on slide, Iran, Gilan Province, near Asalem, 300–600 m a.s.l., large beeches, sifting, 30.VI.1973, leg. A. Senglet, sample 7308. ***Paratypes***: 3 females and 2 males on slide, same data as holotype.

#### Other material.

Iran, female on slide, Gilan Province, Asalem (37°45'N, 48°57'E), leaves and tree holes, sifting, 11.VI.1975, leg. A. Senglet, 7519; female, male and 2 juveniles on slide, Gilan Province, Paresar, tree holes, leaves, sifting, 2.VII.1973, leg. A. Senglet, 7310; male on slide, Mazandaran Province, Nashtarud, forest, reserve, sifting, 10.VII.1973, leg. A. Senglet, 7318; female and 3 males on slide, Mazandaran Province, near Amol, forest, sifting, 18.VII.1973, leg. A. Senglet, 7329b; 4 females, 3 males and juvenile on slide, Mazandaran Province, Aliabad, 30.VII.1974, leg. A. Senglet, 7475; female on slide, Gilan Province, road to Dyavaherdeh, 1100–1300 m a.s.l., 7.VIII. 1974, leg. A. Senglet, 7484.

#### Etymology.

The name of the new species is referring to its exceptionally short macrochaetae Ml.

#### Diagnosis.

Habitus typical of the genus *Deutonura*. Dorsal tubercles present and well developed. 2+2 large pigmented eyes. Buccal cone relatively long and wide, labrum without ogival sclerifications. Head without chaetae E, O and L3. Tubercles Cl and Af separate. No granular area between chaetae A and B on head. Tubercles De on Th. II and III with 3 and 4 chaetae, respectively. Tubercles Di on Abd. V not bilobed. Cryptopygy not developed. Male ventral organ present.

**Figures 42–52.**
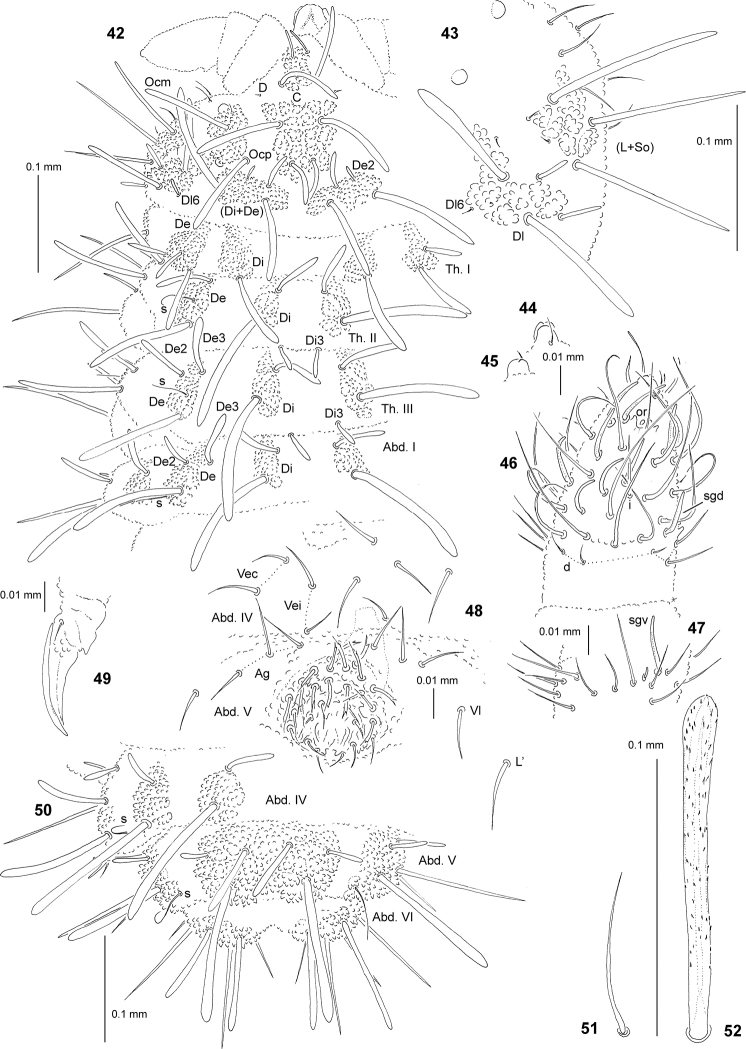
*Deutonura breviseta* sp. nov.: **42** chaetotaxy of head, Th. and Abd. I (holotype), dorsolateral view **43** chaetotaxy of tubercles Dl and (L+So), lateral view **44** apical bulb, dorsal view **45** apical bulb, ventral view **46** dorsal chaetotaxy of Ant. III–IV **47** ventral chaetotaxy of Ant. III **48** ventral chaetotaxy of Abd. IV–V (adult male) **49** claw of leg III, lateral view **50** dorsal chaetotaxy of Abd. IV–VI (holotype) **51** sensillum of Abd. V **52** chaeta Di1 of Abd. V.

#### Description.

General. Body length (without antennae): 0.7 (juvenile) to 1.7 mm (holotype: 0.85 mm). Colour of the body white. 2+2 large black eyes, in a typical arrangement for the genus (Figs 42, 43).

Chaetal morphology. Dorsal ordinary chaetae of five types: long macrochaetae (Ml), short macrochaetae (Mc), very short macrochaetae (Mcc), mesochaetae and microchaetae. Long macrochaetae thickened, slightly arc-like or straight, narrowly sheathed, serrated, apically rounded and extended at apex (Figs 42, 43, 50, 52). Macrochaetae Mc and Mcc morphologically similar to long macrochaetae, but much shorter (Figs 42, 43, 50). Mesochaetae similar to ventral chaetae, thin, smooth and pointed. Microchaetae similar to mesochaetae, but clearly shorter (Figs 42, 43). S-chaetae of terga thin, smooth and short, notably shorter than nearby macrochaetae (Figs 42, 50, 51).

**Table 10.** Chaetotaxy of *Deutonura breviseta* sp. nov.: Cephalic chaetotaxy–dorsal side.

Tubercle	Number of chaetae	Types of chaetae	Names of chaetae
Cl	4	Ml	F
		Mc	G
Af	8	Ml	B
		Mc	A
		mi	C
		mior me	D
Oc	3	Ml	Ocm, Ocp
		mi	Oca
(Di+De)	4	Ml	Di1, De1
		Mc	Di2
		Mcc or mi	De2
Dl	6	Ml	Dl5, Dl1
		Mc	Dl3, Dl4
		Mcc or mi	Dl6
		mi	Dl2
(L+So)	9	Ml	L1, L4, So1
		me	So3–6
		mi	L2, So2

**Table 11.** Chaetotaxy of *Deutonura breviseta* sp. nov.: Chaetotaxy of antennae.

Segment, Group	Number of chaetae	Segment, Group	Number of chaetae adult
I	7	IV	or, 8 S, i, 12 mou, 6 brs, 2 iv
II	12		
III	5 sensilla AO III		
ve	5	ap	8 bs, 5 miA
vc	4	ca	2 bs, 3 miA
vi	4	cm	3 bs, 1 miA
d	5	cp	8 miA, 1 brs

Antennae. Typical of the genus. Dorsal chaetotaxy of Ant. III–IV as in Fig. 46 and Table 11. S-chaetae of Ant. IV of medium length and relatively thin, sensillum sgd short and straight (Fig. 46). Apical vesicle distinct, trilobate (Figs 44, 45). Ventral chaetotaxy of Ant. III as in Fig. 47 and Table 11, ventral chaetotaxy of Ant. IV as Table 11.

Mouthparts. Buccal cone relatively short and wide, labral sclerifications nonogival (Fig. 42). Labrum chaetotaxy: 2/2, 4. Labium with four basal, three distal and four lateral chaetae, papillae x absent. Maxilla styliform mandible thin and tridentate.

Dorsal chaetotaxy and tubercles. Head without granular area between chaetae A and B. Elementary tubercles DE and EE on head absent (Fig. 42). Head without chaetae E, O and L3, chaeta D free (Figs 42, 43). Chaetae Ocm and Ocp of nearly equal length. Chaetae De2 on head usually as Mcc, rarely as mi (Fig. 42). Chaeta Dl6 on head as Mccormi. Th. I with tubercles Di and De not fused. Chaetae Di3 on Th. II–III free. On Th. III, chaetae De2 slightly shorter than De3 (Fig. 42). On Abd. I–III, chaetae De2 distinctly shorter than De3 (Fig. 50). Cryptopygy absent, Abd. VI well visible from above.

Ventral chaetotaxy. On head, groups Vea, Vem and Vep with 3, 4 and 4 chaetae, respectively. Group Vi on head with 6 chaetae. On Abd. IV, furca rudimentary without microchaetae. Male with thick and forked chaetae (male ventral organ) around genital aperture (Abd. V). On Abd. V, chaetae Vl and L’ present (Fig. 48).

Legs. Chaetotaxy of legs as in Table 12. Claw without internal tooth (Fig. 49). On tibiotarsi, chaeta M present and chaetae B4 and B5 relatively long and pointed.

**Table 12.** Chaetotaxy of *Deutonura breviseta* sp. nov.: Postcephalic chaetotaxy.

	Terga				Legs				
	Di	De	Dl	L	Scx2	Cx	Tr	Fe	T
Th. I	1	2	1	–	0	3	6	13	19
Th. II	3	2+s	3+s+ms	3	2	7	6	12	19
Th. III	3	3+s	3+s	3	2	8	6	11	18
					Sterna				
Abd. I	2	3+s	2	3	VT: 4				
Abd. II	2	3+s	2	3	Ve: 5; chaeta Ve1 present				
Abd. III	2	3+s	2	3	Vel: 5; Fu: 5 me, 0 mi				
Abd. IV	2	2+s	3	6	Vel: 4; Vec: 2; Vei: 2; Vl: 4				
Abd. V	(3+3)	5+s			Ag: 3; Vl: 1, L‘: 1				
Abd. VI		7			Ve: 14; An: 2 mi				

#### Remarks.

*Deutonura breviseta* sp. nov. seems to be closest to *D. persica* Smolis, Shayanmehr & Yoosefi-Lafooraki, 2018 recently described from the northern part of Iran (Mazandaran Province, Smolis et al. 2018). However, these species differ in numerous characters, including the number of lateral labial chaetae (in *breviseta*, four, in *persica*, three), the presence of chaetae C on the head (in *breviseta*, present, in *persica*, absent), the number of chaetae (L+So) on the head (in *breviseta*, 9, in *persica* 8), the presence of chaetae Dl3 on the head (in *breviseta*, present, in *persica*, absent), the presence of microchaetae on furca rudimentary (in *breviseta*, absent, in *persica*, present) and the presence of cryptopygy (in *breviseta*, present, in *persica*, absent). Additionally, male ventral organ in *D. breviseta* sp. nov. is built of thickened and forked chaetae on Abd. V only (in *persica*, also on Abd. III, IV and VI).

### Deutonura sengleti sp. nov.

Taxon classificationAnimaliaPoduromorphaNeanuridae

148B3247-C0DD-503B-B4E1-92EF3AB43783

http://zoobank.org/15B48E2F-B8EF-4C46-8A2F-CC09CEDDD62A

Figs 53–61 , Tables 13 , 14 , 15

#### Type material.

***Holotype***: female on slide, Iran, Gilan Province, Shahrbijar, tree hole, humus, sifting, 6.IX.1973, leg. A. Senglet, sample 7366. ***Paratypes***: 2 males on slide, same data as holotype.

#### Other material.

Iran, 2 males on slide, Gilan Province, Limir, large trees in marsh, sifting, 28.VI.1973, leg. A. Senglet, 7306; female, 2 males and juvenile on slide, Gilan Province, road to Jirandeh, 1000 m a.s.l., forest, 9.VIII.1974, leg. A. Senglet, 7486; female, male and juvenile on slide, Gilan Province, near Asalem (37°38'N, 48°48'E), 1800 m a.s.l., tree holes, sifting, 10.VI.1975, leg. A. Senglet, 7516; 2 males and juvenile on slide, Gilan Province, near Asalem (37°40'N, 48°52'E), 1200 m a.s.l., tree holes, sifting, 10.VI.1975, leg. A. Senglet, 7517; female on slide Gilan Province, Asalem (37°45'N, 48°57'E), leaves and tree holes, sifting, 11.VI.1975, leg. A. Senglet, 7519; male on slide, Mazandaran Province, near Amol, forest, sifting, 18.VII.1973, leg. A. Senglet, 7329b; male on slide, Mazandaran Province, road to Tchorteh, 800 m a.s.l., tree and leaves, sifting, 5.VIII.1974, leg. A. Senglet, 7482.

#### Etymology.

The new species is dedicated to Antoine Senglet, collector of the Iranian material studied and prominent Swiss Arachnologist.

#### Diagnosis.

Habitus typical of the genus *Deutonura*. Dorsal tubercles present and well developed. 2+2 large pigmented eyes. Buccal cone relatively long and narrow, labrum without ogival sclerifications. Head without chaetae O, So2 and L3. Tubercles Cl and Af separate. No granular area between chaetae A and B on head. Tubercles De on Th. II and III with 3 and 4 chaetae, respectively. Tubercles Di on Abd. V not bilobed. Cryptopygy not developed. Male ventral organ present.

#### Description.

**Figures 53–61.**
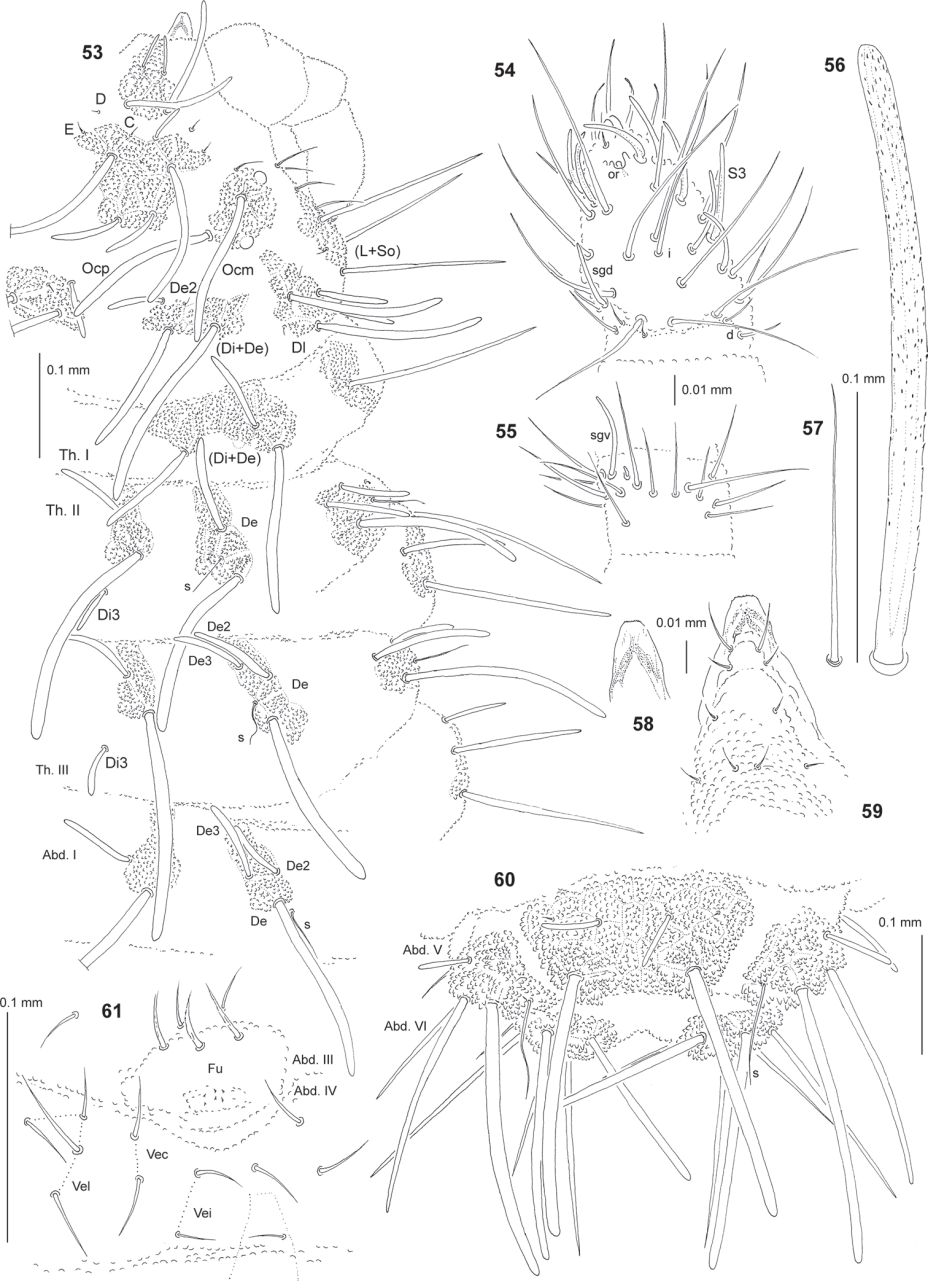
*Deutonura sengleti* sp. nov. **53** chaetotaxy of head, Th. and Abd. I (holotype), dorsolateral view **54** dorsal chaetotaxy of Ant. III–IV **55** ventral chaetotaxy of Ant. III **56** chaeta Di1 of Abd. V **57** sensillum of Abd. V **58** apical part of labrum **59** chaetotaxy and ventral sclerifications of labrum **60** dorsal chaetotaxy of Abd. V–VI (holotype) **61** ventral chaetotaxy of Abd. III–IV (adult male).

General. Body length (without antennae): 0.85 (juvenile) to 1.55 mm (holotype: 1.45 mm). Colour of the body bluish-grey. 2+2 large black eyes, in a typical arrangement for the genus (Fig. 53).

Chaetal morphology. Dorsal ordinary chaetae of five types: long macrochaetae (Ml), short macrochaetae (Mc), very short macrochaetae (Mcc), mesochaetae and microchaetae. Long macrochaetae thickened, slightly arc-like or straight, narrowly sheathed, serrated, cylindrical, apically rounded (Figs 53, 56, 60). Macrochaetae Mc and Mcc morphologically similar to long macrochaetae, but much shorter (Figs 53, 60). Mesochaetae similar to ventral chaetae, thin, smooth and pointed. Microchaetae similar to mesochaetae, but clearly shorter (Figs 53, 60). S-chaetae of terga thin, smooth and short, notably shorter than nearby macrochaetae (Figs 53, 57, 60).

**Table 13.** Chaetotaxy of *Deutonura sengleti* sp. nov.: Cephalic chaetotaxy–dorsal side.

Tubercle	Number of chaetae	Types of chaetae	Names of chaetae
Cl	4	Ml	F
		Mc	G
Af	10	Ml	B
		Mc	A
		Mcc or mi	C
		mi	D, E
Oc	3	Ml	Ocm, Ocp
		mi	Oca
(Di+De)	4	Ml	Di1, De1
		Mc	Di2
		mi or Mcc	De2
Dl	6	Ml	Dl5, Dl1
		Mc	Dl3, Dl4
		mi or Mcc	Dl2
		mi	Dl6
(L+So)	8	Ml	L1, L4, So1
		me	So3–6
		mi or Mcc	L2

Antennae. Typical of the genus. Dorsal chaetotaxy of Ant. III–IV as in Fig. 54 and Table 14. S–chaetae of Ant. IV long and relatively thin, S3 notably longer than others, sensillum sgd of medium size and straight (Fig. 54). Apical vesicle distinct, trilobate. Ventral chaetotaxy of Ant. III as in Fig. 55 and Table 14.

**Table 14.** Chaetotaxy of *Deutonura sengleti* sp. nov.: Chaetotaxy of antennae.

Segment, Group	Number of chaetae	Segment, Group	Number of chaetae adult
I	7	IV	or, 8 S, i, 12 mou, 6 brs, 2 iv
II	12		
III	5 sensilla AO III		
ve	5	ap	8 bs, 5 miA
vc	4	ca	2 bs, 3 miA
vi	4	cm	3 bs, 1 miA
d	5	cp	8 miA, 1 brs

Mouthparts. Buccal cone relatively long and narrow, labral sclerifications nonogival (Figs 58, 59). Labrum chaetotaxy: 4/2, 4 (Fig. 59). Labium with four basal, three distal and four lateral chaetae, papillae x absent. Maxilla styliform mandible thin and tridentate.

Dorsal chaetotaxy and tubercles. Head without granular area between chaetae A and B. Elementary tubercles DE and EE on head absent (Fig. 53). Head without chaetae O, L3 and So2, chaeta D free. Chaetae C as Mccormi (Fig. 53). Chaetae Ocm and Ocp of nearly equal length. Chaetae De2 on head as mior rarely Mcc (Fig. 53). Th. I with tubercles Di and De fused (Fig. 53). Chaetae Di3 on Th. II–III free. On Th. III, chaetae De2 slightly longer than De3 (Fig. 53). On Abd. I–III, chaetae De2 shorter than De3. Cryptopygy absent, Abd. VI well visible from above.

Ventral chaetotaxy. On head, groups Vea, Vem and Vep with 3, 4 and 4 chaetae, respectively. Group Vi on head with 6 chaetae. On Abd. IV, furca rudimentary with 6 minute microchaetae without visible chaetopores (Fig. 61). Male with thick and forked chaetae (male ventral organ) on furca rudimentary (Abd. IV, Fig. 61) and around genital aperture (Abd. V). On Abd. V, chaetae Vl and L’ present.

Legs. Chaetotaxy of legs as in Table 15. Claw without internal tooth. On tibiotarsi, chaeta M present and chaetae B4 and B5 of medium size and pointed.

**Table 15.** Chaetotaxy of *Deutonura sengleti* sp. nov.: Postcephalic chaetotaxy.

	Terga				Legs				
	Di	De	Dl	L	Scx2	Cx	Tr	Fe	T
Th. I	3		1	–	0	3	6	13	19
Th. II	3	2+s	3+s+ms	3	2	7	6	12	19
Th. III	3	3+s	3+s	3	2	8	6	11	18
					Sterna				
Abd. I	2	3+s	2	3	VT: 4				
Abd. II	2	3+s	2	3	Ve: 5; chaeta Ve1 present				
Abd. III	2	3+s	2	3	Vel: 4–5; Fu: 5 me, 6 mi				
Abd. IV	2	2+s	3	6	Vel: 4; Vec: 2; Vei: 2; Vl: 4				
Abd. V	(3+3)	5+s			Ag: 3; Vl: 1, L‘: 1				
Abd. VI		7			Ve: 14; An: 2 mi				

#### Remarks.

The new species runs in the most recent key to *Deutonura* species (Deharveng et al. 2015) to *D. caerulescens* Deharveng, 1982 from France (Deharveng 1982). However, these species differ in the number of chaetae (L+So) on the head (in *sengleti*, 8, in *caerulescens*, 9–10), the presence of microchaetae on furca rudimentary (in *sengleti*, present, in *caerulescens*, absent), the number of chaetae L on Abd. III and IV (in *sengleti*, 3 and 6 chaetae, in *caerulescens*, 4 and 8 chaetae), the number of chaetae on tubercle (De+Dl+L) of Abd. V (in *sengleti*, 5+s, in *caerulescens*, 7+s) and ratio of chaetae Di1:Di2:Di3 on Abd. V (in *sengleti*, 1:4:16, in *caerulescens*, 1:2:4 or 1:3:7).

### Deutonura iranica sp. nov.

Taxon classificationAnimaliaPoduromorphaNeanuridae

0B499BA7-1D6A-5DAB-B3F3-228F3DEF02B8

http://zoobank.org/A3E5E3DA-122E-4C11-888D-CF1265288184

Figs 62–71 , Table 16 , 17 , 18

#### Type material.

***Holotype***: juvenile (second instar) on slide, Iran, West Azerbaijan Province, Choj (38°37'N, 45°02'E), 1.VI.1975, leg. A. Senglet, sample 7503.

#### Etymology.

The species name refers to the country of its collecting.

#### Diagnosis.

Habitus typical of the genus *Deutonura*. Dorsal tubercles present and well developed. 2+2 large pigmented eyes. Buccal cone relatively long and narrow, labrum without ogival sclerifications. Head without chaetae O, So2, L2 and L3. Tubercles Cl and Af separate. No granular area between chaetae A and B on head. Tubercles De on Th. II and III with 3 and 4 chaetae, respectively. Tubercles Di on Abd. V bilobed. Cryptopygy strongly developed.

#### Description.

General. Body length (without antennae): holotype: 1.05 mm. Colour of the body white. 2+2 large black eyes, in a typical arrangement for the genus (Fig. 63).

**Figures 62–71.**
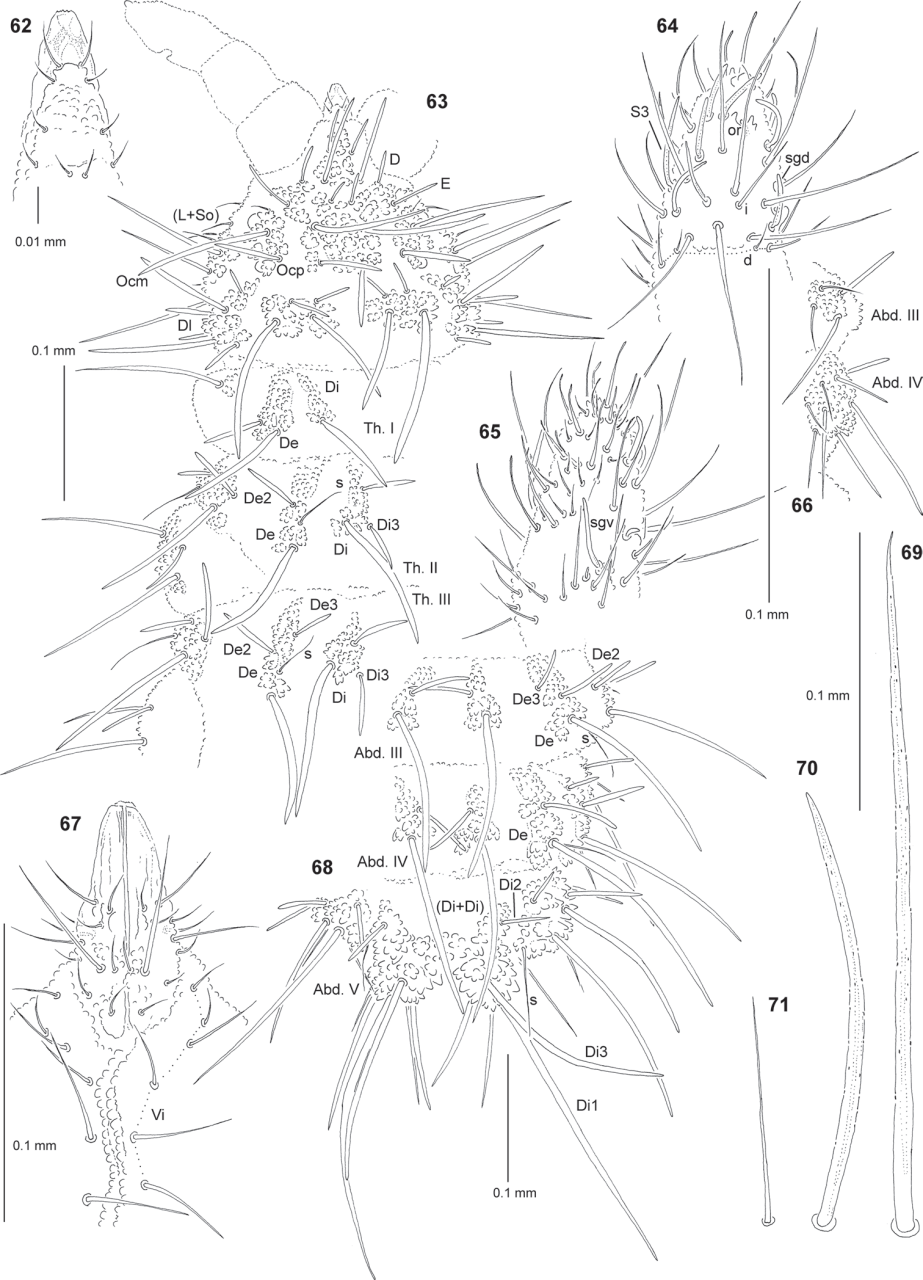
*Deutonura iranica* sp. nov.: **62** chaetotaxy and ventral sclerifications of labrum **63** chaetotaxy of head and Th. (holotype), dorsolateral view **64** dorsal chaetotaxy of Ant. III–IV **65** ventral chaetotaxy of Ant. III–IV **66** chaetotaxy of tubercles L of Abd. III–IV, ventral view **67** chaetotaxy of labium and group Vi**68** dorsal chaetotaxy of Abd. III–VI (holotype) **69** chaeta Di1 of Abd. V **70** chaeta Di2 of Abd. V **71** sensillum of Abd. V.

Chaetal morphology. Dorsal ordinary chaetae of five types: long macrochaetae (Ml), short macrochaetae (Mc), very short macrochaetae (Mcc), mesochaetae and microchaetae. Long macrochaetae relatively thin, arc-like or straight, narrowly sheathed, feebly serrated, apically sharply pointed (Figs 63, 68–70). Macrochaetae Mc and Mcc morphologically similar to long macrochaetae, but much shorter (Figs 63, 68). Mesochaetae similar to ventral chaetae, thin, smooth and pointed. Microchaetae similar to mesochaetae, but clearly shorter. S-chaetae of terga thin, smooth and short, shorter than nearby macrochaetae (Figs 63, 68, 71).

**Table 16.** Chaetotaxy of *Deutonura iranica* sp. nov.: Cephalic chaetotaxy–dorsal side.

**Tubercle**	**Number of chaetae**	**Types of chaetae**	**Names of chaetae**
Cl	4	M	F
		Mc	G
Af	10	Ml	B
		Mc	A, E
		Mcc	C, D
Oc	3	Ml	Ocm
		Mc	Ocp
		mi	Oca
(Di+De)	4	Ml	Di1, De1
		Mcc	Di2, De2
Dl	6	Ml	Dl5, Dl1
		Mc	Dl3, Dl4
		Mcc	Dl2, Dl6
(L+So)	7	Ml	L1, L4, So1
		me	So3–6

Antennae. Typical of the genus. Dorsal chaetotaxy of Ant. III–IV as in Fig. 64 and Table 17. S-chaetae of Ant. IV long and relatively thin, S3 notably longer than others, sensillum sgd of medium size and straight (Fig. 64). Apical vesicle distinct, trilobate. Ventral chaetotaxy of Ant. III–IV as in Fig. 65 and Table 17.

**Table 17.** Chaetotaxy of *Deutonura iranica* sp. nov.: Chaetotaxy of antennae.

Segment, Group	Number of chaetae	Segment, Group	Number of chaetae II instar
I	7	IV	or, 8 S, i, 10 mou, 4 brs, 2 iv
II	12		
III	5 sensilla AO III		
ve	5	ap	8 bs, 5 miA
vc	4	ca	2 bs, 3 miA
vi	4	cm	3 bs, 1 miA
d	5	cp	8 miA, 1 brs

Mouthparts. Buccal cone relatively long and narrow, labral sclerifications nonogival (Figs 62, 67). Labrum chaetotaxy: 4/2, 4 (Fig. 62). Labium with four basal, three distal and four lateral chaetae, papillae x absent (Fig. 67). Maxilla styliform mandible thin and tridentate.

Dorsal chaetotaxy and tubercles. Head without granular area between chaetae A and B. Elementary tubercles DE and EE on head present (Fig. 63). Head without chaetae O, L2, L3 and So2. Chaetae C as Mcc. Chaetae Ocp notably shorter than Ocm. Chaetae De2 on head as Mcc (Fig. 63). Th. I with tubercles Di and De not fused. Chaetae Di3 on Th. II–III connected with tubercle Di. On Th. III, chaetae De2 slightly longer than De3 (Fig. 63). On Abd. I–III, chaetae De2 longer than De3 (Fig. 68). Cryptopygy present and strongly developed, Abd. VI invisible from above (Fig. 68).

Ventral chaetotaxy. On head, groups Vea, Vem and Vep with 4, 3 and 4 chaetae, respectively. Group Vi on head with 6 chaetae (Fig. 67). Tubercles L on Abd. III and IV with 4 and 6 chaetae, respectively (Fig. 66). On Abd. IV, furca rudimentary without microchaetae. On Abd. V, chaetae Vl and L’ present.

Legs. Chaetotaxy of legs as in Table 18. Claw without internal tooth. On tibiotarsi, chaeta M present and chaetae B4 and B5 of medium size and pointed.

**Table 18.** Chaetotaxy of *Deutonura iranica* sp. nov.: Postcephalic chaetotaxy.

	Terga				Legs				
	Di	De	Dl	L	Scx2	Cx	Tr	Fe	T
Th. I	1	2	1	-	0	3	6	13	19
Th. II	3	2+s	3+s+ms	3	2	7	6	12	19
Th. III	3	3+s	3+s	3	2	8	6	11	18
					Sterna				
Abd. I	2	3+s	2	3	VT: 4				
Abd. II	2	3+s	2	3	Ve: 5; chaeta Ve1 present				
Abd. III	2	3+s	2	4	Vel: 5; Fu: 4 me, 0 mi				
Abd. IV	2	2+s	3	8	Vel: 4; Vec: 2; Vei: 2; Vl: 4				
Abd. V	(3+3)	7+s			Ag: 3; Vl: 1, L‘: 1				
Abd. VI		7			Ve: 14; An: 2 mi				

#### Remarks.

Since juveniles (beginning from the first instar) of the subfamily Neanurinae are characterised by the complete chaetotaxy of the head, thorax and abdomen, we decided to describe the new species despite having only one specimen of the second instar. *D. iranica* sp. nov. runs in the most recent key to *Deutonura* species (Deharveng et al. 2015) to *D. gibbosa* Porco, Bedos & Deharveng, 2010, a form common and widespread in southern France (the Alps and Jura), Switzerland, Italy and Slovenia (Porco et al. 2010). Both species are readily distinguished from most members of the genus by the presence of very prominent and conspicuously bilobed tubercle (Di+Di) on the penultimate abdominal segment. This unique character is additionally associated with the specific chaetotaxic arrangement of chaetae Di, with their shift backwards. *D. iranica* sp. nov. can be easily separated from *D. gibbosa* by the presence of white body colour (in *gibbosa* deep to light blue), the presence of 7 chaetae on cephalic tubercle (L+So) (in *gibbosa*, 8–9 chaetae), the presence of cephalic chaetae Ocp equal chaetae A (in *gibbosa*, chaetae Ocp distinctly longer than A) and the presence of 4 lateral labial chaetae (in *gibbosa*, 3 chaetae).

### Paravietnura rostrata sp. nov.

Taxon classificationAnimaliaPoduromorphaNeanuridae

FBFBEB25-68F5-52EE-982B-F10D766B149F

http://zoobank.org/E4B57858-235D-4FCC-99D6-A7AFBE6848A7

Figs 72–82 , Tables 19 , 20 , 21

#### Type material.

***Holotype***: juvenile (second instar) on slide, Iran, Gilan Province, Shahrbijar, tree hole, humus, sifting, 6.IX.1973, leg. A. Senglet, sample 7366.

#### Etymology.

The name of the new species referring to its exceptionally-long buccal cone.

#### Diagnosis.

Habitus typical of the genus *Paravietnura* with stumpy and short body. Macrochaetae long thick and widely sheathed. 2+2 large pigmented eyes. Buccal cone extremely long and narrow, labrum with ogival sclerifications. Tubercle (Af + 2Oc) with chaetae B and Ocm, chaetae A and Ocp absent. Tubercle Cl without chaetae G. Tubercle (Dl+L+So) with 9 chaetae. Furca rudimentary with minute and difficult microchaetae, without chaetopores.

#### Description.

General. Body length (without antennae): holotype: 0.45 mm. Colour of the body bluish. 2+2 large black eyes, in a typical arrangement for the genus (Fig. 75).

**Figures 72–82.**
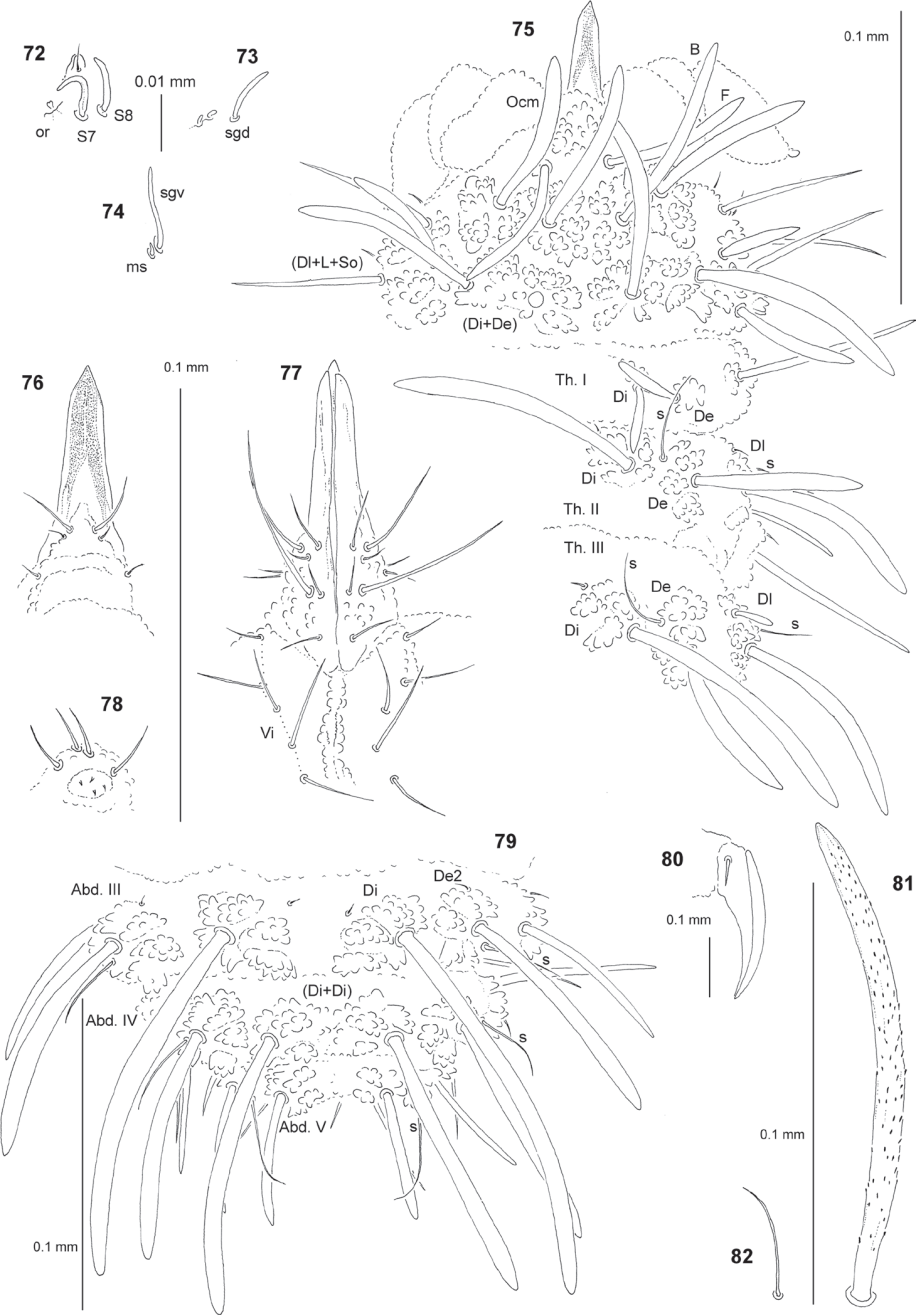
*Paravietnura rostrata* sp. nov.: **72** apical part of Ant. IV, dorsal view **73** sensillum sgd and microsensilla of AOIII, dorsolateral view **74** sensillum sgv and microsensillum of Ant. III **75** chaetotaxy of head and Th. (holotype), dorsolateral view **76** chaetotaxy and ventral sclerifications of labrum **77** chaetotaxy of labium and group Vi**78** furca rudimentary **79** dorsal chaetotaxy of Abd. III–VI (holotype) **80** claw of leg III, lateral view **81** chaeta Di1 of Abd. III **82** sensillum of Abd. IV.

Chaetal morphology. Dorsal ordinary chaetae of five types: long macrochaetae (Ml), short macrochaetae (Mc), very short macrochaetae (Mcc), mesochaetae and microchaetae. Long macrochaetae thickened, arc-like, widely sheathed, strongly serrated, apically rounded (Figs 75, 79, 81). Macrochaetae Mc and Mcc morphologically similar to long macrochaetae, but much shorter (Figs 75, 79). Mesochaetae similar to ventral chaetae, thin, smooth and pointed. Microchaetae similar to mesochaetae, but clearly shorter (Figs 75, 79). S–chaetae of terga thin, smooth and short, shorter than nearby macrochaetae (Figs 75, 79, 82).

**Table 19.** Chaetotaxy of *Paravietnura rostrata* sp. nov.: Cephalic chaetotaxy–dorsal side.

Tubercle	Number of chaetae	Types of chaetae	Names of chaetae
Cl	2	Ml	F
(Af+2Oc)	4	Ml	B, Ocp
(Di+De)	2	Ml	Di1, De1
(Dl+L+So)	9		impossible to recognise

Antennae. Typical of the genus. Dorsal and ventral chaetotaxy of Ant. III–IV as in Figs 72–74 and Table 20. S-chaetae of Ant. IV relatively short and thin (Fig. 72), sensillum sgd of medium size and straight (Fig. 73), sensillum sgv relatively long and slightly s-shaped (Fig. 74). Apical vesicle distinct, bilobate (Fig. 72).

**Table 20.** Chaetotaxy of *Paravietnura rostrata* sp. nov.: Chaetotaxy of antennae.

Segment, Group	Number of chaetae	Segment, Group	Number of chaetae II instar
I	7	IV	or, 8 S, i, 10 mou, 4 brs, 2 iv
II	11		
III	5 sensilla AO III		
ve	5	ap	8 bs, 5 miA
vc	4	ca	2 bs, 3 miA
vi	4	cm	3 bs, 1 miA
d	4	cp	8 miA, 1 brs

Mouthparts. Buccal cone extremely elongated with labral sclerifications ogival (Figs 76, 77). Labrum chaetotaxy: 0/2, 4, without prelabral chaetae (Fig. 76). Labium with three basal, three distal and two lateral chaetae, papillae x absent (Fig. 77). Maxilla styliform mandible thin and tridentate.

Dorsal chaetotaxy and tubercles. Chaetotaxy of head as in Fig. 75 and Table 19. Chaetotaxy of Th. and Abd. As in Figs 75, 79 and Table 21. On Th. I, tubercle De with one chaeta (Fig. 75). On Th. II and III, chaetae Di 3 absent. Th. II and III with two chaetae De (Fig. 75). On Abd. IV, chaetae Di1 distinctly longer than Abd. V (Fig. 79). On Abd. V, chaetae Di2 and Di3 absent. Tubercle Di of Abd. IV partially fused (Fig. 79). Cryptopygy present and strongly developed, Abd. VI invisible from above (Fig. 79).

Ventral chaetotaxy. On head, groups Vea, Vem and Vep with 3, 2 and 4 chaetae, respectively. Group Vi on head with 5 chaetae (Fig. 77). On Abd. IV, furca rudimentary with 4 minute microchaetae and 4 mesochaetae (Fig. 78). On Abd. V, chaetae Vl present and L’ absent.

Legs. Chaetotaxy of legs as in Table 21. Claw without internal tooth (Fig. 80). On tibiotarsi, chaeta M present and chaetae B4 and B5 of medium size and pointed.

**Table 21.** Chaetotaxy of *Paravietnura rostrata* sp. nov.: Postcephalic chaetotaxy.

	Terga				Legs				
	Di	De	Dl	L	Scx2	Cx	Tr	Fe	T
Th. I	1	1	1	–	0	3	6	13	19
Th. II	2	1+s	2+s+ms	3	2	7	6	12	19
Th. III	2	1+s	2+s	3	2	8	6	11	18
					Sterna				
Abd. I	2	2+s	2	2	VT: 4				
Abd. II	2	2+s	2	2	Ve: 3; chaeta Ve1 present				
Abd. III	2	2+s	2	2	Vel: 3; Fu: 4 me, 4 mi				
Abd. IV	(1+1)	1+s	3	3	Vel: 2; Vec: 2; Vei: 2; Vl: 4				
Abd. V	4+s				Ag: 2; Vl: 1				
Abd. VI		7			Ve: 11; An: 1mi				

#### Remarks.

No doubt, the new species is the third member of the remarkable Neanurinae genus *Paravietnura* Smolis & Kuznetsova, 2018 described recently from the Caucasus (Smolis and Kuznetsova 2018). *Paravietnura rostrata* sp. nov. seems to be the closest to *P. notabilis* Smolis & Kuznetsova, 2018; however, it can be easily separated from the mentioned species because of the reduction of its cephalic chaetotaxy (in *rostrata*, chaetae G and Ocp absent, in *notabilis*, present), extremely elongated labrum, which is well visible from above (in *notabilis*, feebly elongated and practically invisible from above), absence of prelabral chaetae (in *notabilis*, 2 chaetae present), the presence of 1+1 chaetae De on Th. I (in *notabilis*, 2+2 chaetae present), the absence of chaetae Di3 on Th. (in *notabilis*, present), reduction of the number of chaetae De on Th. II and III (in *rostrata*, 1+s chaetae, in *notabilis*, 2+s and 3+s chaetae, respectively), the absence of chaetae De2 and De3 on Abd. I–III (in *notabilis*, present), the fusion of tubercles Di on Abd. IV (in *notabilis*, not fused) and the presence of 1 chaeta Di on Abd. V (in *notabilis*, 3 chaetae Di present).

##### New Records

### Cryptonura maxima

Taxon classificationAnimaliaPoduromorphaNeanuridae

Smolis, Falahati & Skarżyński, 2012

60F1401A-46E8-548B-A8CC-EC165051640C

#### Material.

Iran, Mazandaran Province, Baladeh, 2200 m a.s.l., 12.VII.1974, leg. A. Senglet, sample 7459; numerous specimens on slide, Iran Mazandaran Province, Aliabad, 30.VII.1974, leg. A. Senglet, 7475.

#### Note.

Up to date, the species was known from the Elburz Mts. in Golestan Province (Smolis et al. 2012).

### Cryptonura persica

Taxon classificationAnimaliaPoduromorphaNeanuridae

Smolis, Falahati & Skarżyński, 2012

AFDC7E5C-B0D9-55D2-A37F-397E76621E71

#### Material.

Iran, Mazandaran Province, near Gorgan, forest, mosses, sifting, 20.VII.1973, leg. A. Senglet, sample 7332; Mazandaran Province, near Shahpasand, leaves, sifting, 29.VII.1974, leg. A. Senglet, 7473; West Azerbaijan Province, Choj (38°37'N, 45°02'E), 1.VI.1975, leg. A. Senglet, 7503; Golestan Province, near Tangrah (37°23'N, 55°50'E), 16.VII.1975, leg. A. Senglet, 7552; North Khorasan Province, near Tangrah (37°20'N, 56°01'E), 16.VII.1975, leg. A Senglet, 7553; Golestan Province, near Loveh (37°20'N, 55°44'E / 700 m a.s.l.), 21.VIII.1975, leg. A. Senglet, 7572; Golestan Province, near Loveh (37°18'N, 55°43'E / 1200 m a.s.l.), 21.VIII.1975, leg. A Senglet, 7573; Semnan Province, near Loveh (37°19'N, 55°46'E / 1300 m a.s.l.), 22.VIII.1975, leg. A. Senglet, 7574.

#### Note.

Similarly to the previous species, *C. persica* was known exclusively from the Elburz Mts. in Golestan Province (Smolis et al. 2012). The outlined records, from provinces West Azerbaijan, Mazandaran, Semnan and North Khorasan, shows that this form seems to be quite common and widespread in north-western Iran.

### Deutonura persica

Taxon classificationAnimaliaPoduromorphaNeanuridae

Smolis, Shayanmehr & Yoosefi-Lafooraki, 2018

D09450A7-F45E-5FEA-B42F-6CB08643DF14

#### Material.

Iran, Gilan Province, near Asalem (37°42'N, 48°53'E), 450 m a.s.l., tree holes, sifting, 10.VI.1975, leg. A. Senglet, sample 7518; Iran, Mazandaran Province, Ivel (36°14'N, 53°37'E / 1500 m a.s.l.), under stones, 11.VII.1975, leg. A Senglet, 7547A.

#### Note.

Until now, the species was known from its type locality only: Hezarjarib Forest in region Neka in Mazandaran Province (Smolis et al. 2018).

### Endonura longirostris

Taxon classificationAnimaliaPoduromorphaNeanuridae

Smolis, Shayanmehr, Kuznetsova & Yoosefi-Lafooraki, 2017

80C7150E-9F51-56D0-8917-C932DDA54A04

#### Material.

Iran, Mazandaran Province, Nashtarud, forest, reserve, sifting, 10.VII.1973, leg. A. Senglet, sample 7318; Iran, Mazandaran Province, near Delaam, forest, 4.VIII.1974, leg. A. Senglet, 7478; Golestan Province, near Loveh (37°20'N, 55°44'E / 700 m a.s.l.), 21.VIII.1975, leg. A. Senglet, 7572.

#### Note.

Up to now, this very characteristic member of the genus *Endonura* was known from two localities in Mazandaran Province (Smolis et al. 2017).

### Endonura paracentaurea

Taxon classificationAnimaliaPoduromorphaNeanuridae

Smolis, Shayanmehr, Kuznetsova & Yoosefi-Lafooraki, 2017

2FD81BE4-C854-52F8-9258-4FADF32EB08D

#### Material.

Iran, Gilan Province, Limir, ;large trees in marsh, sifting, 28.VI.1973, leg. A. Senglet, sample 7306; Gilan Province, Shahrbijar, tree hole, humus, sifting, 6.IX.1973, leg. A. Senglet, 7366; Mazandaran Province, road to Tchorteh, 800 m a.s.l., tree and leaves, sifting, 5.VIII.1974, leg. A. Senglet, 7482.

#### Note.

Until now, *Endonura paracentaurea* was recorded exclusively from Mazandaran Province (Smolis et al. 2017).

### Neanura deharvengi

Taxon classificationAnimaliaPoduromorphaNeanuridae

Smolis, Shayanmehr & Yoosefi-Lafooraki, 2018

02BC902A-14A3-591A-8CC1-F5284DDACBA1

#### Material.

Iran, Gilan Province, Limir, big trees in marsh, sifting, 28.VI.1973, leg. A. Senglet, sample 7306; Mazandaran Province, Nashtarud, forest, reserve, sifting, 10.VII.1973, leg. A. Senglet, 7318; Mazandaran Province, Kiasar, very dry forest, sifting, 22.VII.1973, leg. A. Senglet, 7334.

#### Note.

To date, this unique member of the genus *Neanura* MacGillivray, 1893 characterised by strong reduction of cephalic chaetotaxy, was recorded from two localities in Mazandaran Province only (Smolis et al. 2018).

### Neanura muscorum

Taxon classificationAnimaliaPoduromorphaNeanuridae

(Templeton, 1835)

4071A517-C817-551F-ACE4-744E97A5AF78

#### Material.

Iran, Gilan Province, Zandżan (36°43'N, 48°21'E), 15.IX.1973, leg. A. Senglet, sample 7372.

#### Note.

Up to now, this cosmopolitan and the most widespread member of the subfamily Neanurinae was recorded from three Iranian provinces: Zanjan, Gilan and Mazandaran (Cox 1982, Yahyapour 2012).

### Protanura papillata

Taxon classificationAnimaliaPoduromorphaNeanuridae

Cassagnau & Delamare Deboutteville, 1955

DE62A109-DC0F-5945-8EE4-87F3CF6B6449

#### Material.

Iran, Kermanshah Province, Geravand, 5.VIII.1973, leg. A. Senglet, sample 7344.

#### Note.

This species is known from Lebanon, Israel and Iran (Smolis et al. 2016b). The present record is the third from Kermanshah Province.

## Discussion

Until recently, the whole knowledge on richness and diversity of Iranian Neanurinae was based solely on a Cox’s (1982) paper, in which four European and rather common taxa, i.e. *Neanura muscorum* and *Bilobella aurantiaca* (Caroli, 1912) were mentioned. However, the last decade has resulted in a real explosion of research on Iranian Collembola. Taking into account all recent data, one can conclude that Neanurinae fauna of Iran contains 21 species of the following genera: *Bilobella* Caroli, 1912 – 1; *Cryptonura* Cassagnau, 1979 – 2; *Deutonura* Cassagnau, 1979 – 5; *Endonura* Cassagnau, 1979 – 8; *Neanura* MacGilliwray, 1893 – 2; *Paravietnura* Smolis & Kuznetsova, 2018 – 1; *Persanura* Mayvan, Smolis & Skarżyński, 2015 – 1; *Protanura* Börner, 1906 and *Thaumanura* Börner, 1932 – 1 (Cox 1982; Smolis et al. 2012; Mayvan et al. 2015b; Smolis et al. 2016a, b, 2017; Smolis and Kuznetsova 2018; Smolis et al. 2018). Despite the fact that the image of diversity and richness of Iranian Neanurinae is still incomplete, some general comments can be made.

Firstly, the Iranian fauna is characterised by a remarkable percentage of endemites, since seventeen species are known exclusively from this country. This number is probably underestimated as earlier records of some taxa, i.e. *Bilobella aurantiaca*, *Thaumanura echinata* (Kos, 1940) and *Deutonura decolorata* (Gama & Gisin, 1964 in: Gisin 1964) are rather unlikely and should be revised. Such a high number of endemites is certainly noteworthy; nevertheless, it is a known and rather general phenomenon for this group of springtails. Research conducted, both in tropical and temperate forests, indicated that Neanurinae have a strong tendency to speciation and their fauna on a larger geographical scale is often characterised by a high degree of endemism (e.g. Deharveng 1979; Cassagnau and Palacios-Vargas 1983; Deharveng and Weiner 1984; Cassagnau and Deharveng 1984; Cassagnau 1988, 1996; Greenslade 1994; Palacios-Vargas and Simón Benito 2007; Janion et al. 2011; Queiroz and Deharveng 2015; Smolis 2017).

Secondly, in terms of species richness, this fauna should be treated even today as very rich. Especially, the Hyrcanian forest, where sixteen species of the subfamily were noted, seems to be not only a national but also a regional hot spot. The observed situation, however, may not be especially surprising as this huge and diversified area covers almost one million hectares and ranges from west to east through five Iranian Provinces: Ardabil, Gilan, Mazandaran, Golestan and North Khorasan. In addition, this forest is a worldwide and commonly-known refuge for many iconic and spectacular mammals, i.e. the Persian leopard *Panthera pardus ciscaucasica*, trees, i.e. the Persian ironwood *Parrotia persica*, the Caspian locust tree *Gleditsia capsica* and insects, i.e. the longhorn beetle *Parandra caspia*, the red flat beetle *Cucujus muelleri* (e.g. Sagheb-Talebi et al. 2014; Mayvan et al. 2015a; Müller et al. 2015; Bussler 2017).

Finally, current and especially future knowledge (many regions of Iran still remain unexplored, see Shayanmehr et al. 2013, Fig. 2) of the Iranian Neanurinae fauna could shed light on key issues such as its origin and relationship with fauna of neighbouring regions. For example, the similarity of Iranian fauna to that of the Caucasus (presence of genera *Paravietnura* and *Persanura*) and the east Mediterranean region (presence of *Protanura papillata* and genus *Cryptonura*) should already be underlined.

## Supplementary Material

XML Treatment for Endonura agnieskae

XML Treatment for Endonura annae

XML Treatment for Endonura schwendingeri

XML Treatment for Deutonura breviseta

XML Treatment for Deutonura sengleti

XML Treatment for Deutonura iranica

XML Treatment for Paravietnura rostrata

XML Treatment for Cryptonura maxima

XML Treatment for Cryptonura persica

XML Treatment for Deutonura persica

XML Treatment for Endonura longirostris

XML Treatment for Endonura paracentaurea

XML Treatment for Neanura deharvengi

XML Treatment for Neanura muscorum

XML Treatment for Protanura papillata
